# Exploration of Free Energy Surface of the Au_10_ Nanocluster at Finite Temperature

**DOI:** 10.3390/molecules29143374

**Published:** 2024-07-18

**Authors:** Francisco Eduardo Rojas-González, César Castillo-Quevedo, Peter Ludwig Rodríguez-Kessler, José Oscar Carlos Jimenez-Halla, Alejandro Vásquez-Espinal, Rajagopal Dashinamoorthy Eithiraj, Manuel Cortez-Valadez, José Luis Cabellos

**Affiliations:** 1 Departamento de Física, Edificio 3F, Universidad de Sonora, Hermosillo 83000, Sonora, Mexico; francisco.rojas@unison.mx; 2 Departamento de Fundamentos del Conocimiento, Centro Universitario del Norte, Universidad de Guadalajara, Carretera Federal No. 23, km. 191, Colotlán 46200, Jalisco, Mexico; castillo.quevedo@cunorte.udg.mx; 3 Centro de Investigaciones en Óptica, A.C. (CIO) Lomas del Bosque 115, León 37150, Guanajuato, Mexico; plkessler@cio.mx; 4 Departamento de Química, División de Ciencias Exactas y Naturales, Universidad de Guanajuato, Noria Alta s/n, Guanajuato 36050, Guanajuato, Mexico; jjimenez@ugto.mx; 5 Química y Farmacia, Facultad de Ciencias de la Salud, Universidad Arturo Prat. Casilla 121, Iquique 1100000, Chile; alvasquez@unap.cl; 6 Department of Physics, School of Advanced Sciences, Vellore Institute of Technology (VIT), Chennai 600 127, Tamil Nadu, India; eithiraj.rd@vit.ac.in; 7 CONAHCYT-Departamento de Investigación en Física, Universidad de Sonora, Apdo. Postal 5-88, Hermosillo 83190, Sonora, Mexico; manuelcortez@live.com; 8 Coordinación de Investigación y Desarrollo Tecnológico, Universidad Politécnica de Tapachula, Carretera Tapachula a Puerto Madero km. 24, Tapachula 30830, Chiapas, Mexico

**Keywords:** global minimum, Au cluster, density functional theory, temperature, Boltzmann factors, Gibbs free energy, entropy, enthalpy, thermochemistry, nanothermodynamics, IR spectra, quantum statistical mechanics, genetic algorithm, chemical bonding analysis, effects relativistic, zero-order regular approximation, adaptive natural density partitioning, QTAIM, DLPNO-CCSD(T)

## Abstract

The first step in comprehending the properties of Au_10_ clusters is understanding the lowest energy structure at low and high temperatures. Functional materials operate at finite temperatures; however, energy computations employing density functional theory (DFT) methodology are typically carried out at zero temperature, leaving many properties unexplored. This study explored the potential and free energy surface of the neutral Au_10_ nanocluster at a finite temperature, employing a genetic algorithm coupled with DFT and nanothermodynamics. Furthermore, we computed the thermal population and infrared Boltzmann spectrum at a finite temperature and compared it with the validated experimental data. Moreover, we performed the chemical bonding analysis using the quantum theory of atoms in molecules (QTAIM) approach and the adaptive natural density partitioning method (AdNDP) to shed light on the bonding of Au atoms in the low-energy structures. In the calculations, we take into consideration the relativistic effects through the zero-order regular approximation (ZORA), the dispersion through Grimme’s dispersion with Becke–Johnson damping (D3BJ), and we employed nanothermodynamics to consider temperature contributions. Small Au clusters prefer the planar shape, and the transition from 2D to 3D could take place at atomic clusters consisting of ten atoms, which could be affected by temperature, relativistic effects, and dispersion. We analyzed the energetic ordering of structures calculated using DFT with ZORA and single-point energy calculation employing the DLPNO-CCSD(T) methodology. Our findings indicate that the planar lowest energy structure computed with DFT is not the lowest energy structure computed at the DLPN0-CCSD(T) level of theory. The computed thermal population indicates that the 2D elongated hexagon configuration strongly dominates at a temperature range of 50–800 K. Based on the thermal population, at a temperature of 100 K, the computed IR Boltzmann spectrum agrees with the experimental IR spectrum. The chemical bonding analysis on the lowest energy structure indicates that the cluster bond is due only to the electrons of the 6 s orbital, and the Au d orbitals do not participate in the bonding of this system.

## 1. Introduction

Nanoclusters of transition metals enhance catalytic activity due to their high surface-to-volume ratio and high surface energy [1,2]. Moreover, the transition metal atoms have incompletely filled d orbitals, allowing them to easily donate and accept electrons from other ions [3,4]. Gold is the most electronegative metallic atom [5], and when it forms bulk, gold is chemically inert [6,7,8,9]. However, when gold atoms make small clusters at nanoscale size, gold is not a noble, nonreactive material [10]. The Au clusters find applications in many fields of chemistry, physics, and nanomaterials [11]. Gold nanoclusters with diameters below 10 nm [6] are highly catalytic active due to quantum size effects [7,12], high oxidation states [13,14], and low coordinated atoms [15]., e.g., supported gold nanoclusters catalyze CO oxidation at low temperatures [8,16].

The lowest energy configurations of neutral Au clusters have been investigated from experimental and theoretical perspectives [11,17]. From a theoretical approach, there is a need to determine the cluster size at which the transition from 2D to 3D occurs [8,11,18], considering relativistic corrections, van der Waals forces, and temperature. This paper aims to provide insight in this direction.

In a study by Bravo-Pérez et al. [19], the neutral Au*_n_* (*n* = 3–7) clusters were investigated using density functional theory (DFT), and it was found that the lowest energy structure is planar. From an experimental standpoint, there is evidence that small Au clusters up to *n* = 7 are planar [20]. By using scalar-relativistic pseudopotentials for the valence electrons of gold and employing DFT, the lowest energy structure of Au*_n_* (*n* = 2–10) anionic and neutral clusters were located, revealing 3D structures [21]. Previous research has also looked into the lowest-energy structures of Au_*n*_ (*n* = 2–20) clusters using a genetic algorithm coupled with DFT and empirical potential; the authors found that the low-energy structures are planar. Moreover, the 2D–3D transitions take place at Au atomic clusters with seven atoms [22]. For neutral Au clusters, previous high-level ab-initio calculations show that a structural transition from 2D planar to 3D structures occurs within the size range *n* = 7–10 [23,24,25,26,27]. Moreover, the transition could be between 12 and 15 atoms [28,29]. Assadollahzadeh et al. [30] reported a systematic search for the lowest energy structures of Au*_n_* (*n* = 2–20) clusters employing a genetic algorithm coupled to DFT with a relativistic pseudopotential. They found planar structures up to a cluster size of Au_10_. Sarosi et al. [31] reported a planar putative lowest energy structure employing DFT at the level of theory B3PW91/def2-TZVPD. Recently, Pham Vu Nhat et al. [11] suggested that the 2D–3D transitions for Au clusters occur at the size Au_10_ cluster. However, previous studies on the structure, stability, and bonding in Au_10_ clusters reported the putative global minimum as the three-dimensional structure at the MP2 level of theory [32]. The 2D–3D transition in neutral Au*_n_* clusters depends on the level of theory employed [31] and temperature. Furthermore, to establish a close-real value of energies, we suggest that energies must be computed at a single-point CCSD(T) level of theory [33,34].

The chemistry and physics of gold are strongly dominated by relativistic effects [35], e.g., the Au–Au bonding distance is shortened due to relativistic effects. Previous studies on the structure of small, neutral gold clusters employed the ZORA as a relativistic correction [10,36]. Romaniello et al. [37] included relativistic effects for calculating the linear response properties of metals, particularly the Au atom. Soto et al. [38] employed ZORA approximation to study the gold cluster cation Au_7_, and they found a putative lowest-energy puckered 3D structure. Due to relativistic effects, the preference for the planar shape of neutral gold clusters continues up to 11 atoms [27,30,39].

The van der Waals interactions (vdW) are critical for predicting the stability of molecules [40,41,42] and can change the energetic ordering of isomers [32,34,40,43,44]. Previous studies on small gold clusters in the size range of 12–14 atoms found that, considering van der Waals interactions in the energy calculations, the 2D–3D transition size is 13 atoms [45]. Besides relativistic effects and vdW interactions, the temperature is a critical factor in all molecular systems, mainly transition-metal nanoclusters exhibit catalytic activity at high temperatures. Gold nanoclusters, specifically, have recently received attention because of the discovery of their catalytic activity at low temperatures [10].

The experiments are performed at finite temperatures [10,46,47], and the materials also work at finite temperatures [32,34,44,47]. However, from the theoretical point of view, DFT calculations are typically performed at zero temperature and are valid at zero temperature and high vacuum conditions. However, using statistical mechanics, we can include the effects of temperature and pressure. In the mid-1960s, Mermin et al. [48] studied thermal properties in an inhomogeneous electron gas. Previous works have also addressed temperature in DFT [49,50]. More recently, DFT has been developed to account for finite temperatures [51,52,53]. Currently, temperature effects can be considered using two methods. The first is ab initio molecular dynamics, in which temperature is controlled by a thermostat [54,55]. Second, employing quantum statistical mechanics that requires the vibrational modes or phonon spectrum to compute the molecular partition function that contains all thermodynamic information [32,34,56,57,58]. A change in energetic separation among isomers distribution and dynamic structural rearrangements are the first effects of temperature [10,34,41,59]. Particularly in small gold clusters, the transitions from 2D to 3D structure depend on temperature. However, few studies have examined the gold isomers’ stability at finite temperatures [59]. Goldsmith et al. [41] studied neutral gas gold clusters at finite temperatures by replica-exchange ab initio molecular dynamics. A previous study on neutral Au_12_ predicts the dynamic coexistence of multiple planar and non-planar structures at room temperature [60]. Ghiringhelli et al. [10] studied the structure of small neutral gold clusters Au_3_, Au_4_, and Au_7_ and their infrared spectra. Previous work observed the IR spectra in small neutral gold clusters containing up to eight Au atoms and compared them with computed IR spectra [61,62]. In previous research on chemical bonding in Au clusters, Rodríguez et al. [63] used the QTAIM scheme to calculate the atomic properties and determine the chemical bonding at the Au-gold–thiol interface. Recently, Chebotaev et al. [64] studied the interactions between pterin and gold clusters using QTAIM theory. Additionally, Zubarev et al. [65] used the AdNDP method to study chemical bonding in Au_20_ cluster.

In this paper, we utilized a genetic algorithm coupled with DFT using the version 5.0 of the ORCA quantum chemistry program suite [66] to investigate the potential and free energy surfaces of the Au_10_ cluster. Our approach included local optimizations with the ZORA relativistic approximation and the dispersion D3BJ [67], considering thermal effects for temperatures ranging from 50 to 800 K based on quantum statistical mechanics. By employing a Boltzmann weights scheme [33,34,43,44], we computed the temperature-dependent Boltzmann–IR spectrum utilizing the calculated thermal population. Our study successfully validated that the computed Boltzmann–IR spectrum aligns with IR experimental data. Notably, we identified the 2D elongated hexagon configuration as the probable global minimum within the temperature range of 50–800 K, indicating that the transition from 2D to 3D does not occur in the Au_10_ cluster. Additionally, we rigorously examined the energetic ordering of low-energy structures computed at the DFT level against single-point energy calculations using the domain-based local pair natural orbital coupled cluster method with single-, double-, and perturbative triple-excitations (DLPNO-CCSD(T)) level of theory.

The remaining sections of this manuscript are organized: Section 2 presents an overview of the theory and computational details. In Section 3, we delve into the results covering various aspects such as the low-energy structures considering ZORA and DBJ3 dispersion, the chemical bonding with AdNDP and QTAIM, the energetic ordering of isomers computed at the DLPNO-CCSD(T) level of theory, the computed IR spectrum temperature dependent and compared with observed data, and the thermal population. These findings culminate in the conclusions presented in Section 4.

## 2. Results and Discussion

### 2.1. The Lowest Energy Structures

**Figure 1:**
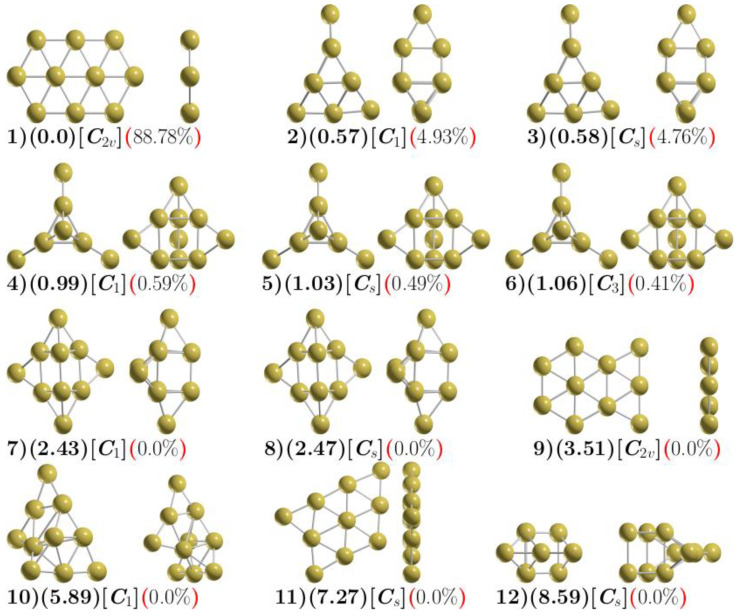
The optimized geometries of the neutral Au_10_ cluster at PBE0 ZORA SARC-ZORA-TZVP SARC/J D3BJ level of theory. The most important low-energy isomers appear in two orientations. The relative Gibbs free energies (in kcal/mol) are shown in parentheses, the point group symmetry is displayed in square brackets, while the thermal population at 100 K is given in red parentheses. The yellow spheres stand for the Au atoms (atomic coordinates available in Supplementary Materials).

The final optimized geometries depicted in Figure 1 are reported at PBE0-D3BJ [67,68] and ZORA [10,36] SARC-ZORA-TZVP [69] SARC/J [70] and employing AutoAux generation procedure [71] level of theory. All calculations were performed using the version 5.0 of the ORCA quantum chemistry program suite [72].

Figure 1 shows the 12 most energetically stable structures of Au_10_. The relative Gibbs free energies are computed at 100 K, 1 atm, and lying within the energy range of 0.0–8.6 kcal/mol. The harmonic vibrational analysis of these structures showed no imaginary frequencies, corroborating that they are energy minima. The putative global minimum, **1**, can be described as a planar elongated hexagon formed by triangular units with symmetry *C*_2*v*_. The thermal population is 88.7%, and the average Au–Au bond length is 2.7 Å. This value is slightly longer than the experimental one, 2.4715 Å, obtained from high-resolution rotational spectroscopy in the Au dimer [17,73] This system, **1**, has been reported as the putative global minimum by different authors [31].

The second and third isomers lie around 0.57 kcal/mol above **1** in Gibbs free energy. These structures resemble a trigonal prism capped by three and one atoms at both ends. Their corresponding thermal populations are 4.93% and 4.76%, respectively.

The next series of isomers, **4**, **5**, and **6** are located at 0.99–1.06 kcal/mol above the putative global minimum. Their corresponding thermal population are 0.59, 0.49, and 0.41%. This form is quite similar to that found in low-lying Sn-type clusters [74]. This isomer is similar with what was previously reported as low-lying energy structures [11].

The two following isomers, **7** and **8**, lie at 2.43 and 2.47 kcal/mol higher in energy than **1**, and they are distorted capped trigonal prisms with a zero thermal population. Nhat et al. [11] used the PNO-LCCSD(T)-F12/aug-cc-pVTZ-PP level of theory, plus ZPE energy correction based on PBE/cc-pVTZ-PP-optimized geometries. They found the capped trigonal prism at 1.8 kcal/mol above the trigonal edge-capped prism. Structures **9** and **11** (3.51 and 7.27 kcal/mol above **1**) have been reported as quasi-planar at 300 K [41]. The former is located at 3.3 kcal/mol at the PNO-LCCSD(T)-F12/aug-cc-pVTZ-PP level of theory [11]. The distorted structure, **10**, at 5.89 kcal/mol, is a folded flake structure that has not been reported. The symmetric structure, 12, at 8.59 kcal/mol, is a small distorted cage.

To explore spin effects in determining the lowest energy structure of Au_10_, we computed the triplet state. All neutral Au_10_ optimized with spin multiplicities singlets were taken as initial structures for optimization without any symmetry restriction being performed considering triplet states. Our results suggest that the lowest-lying structure in the triplet state is 30 kcal/mol above the singlet putative global minimum. The second low-energy triplet is at 33 kcal/mol. Thus, higher spin states (up to triplet) are not energetically favored.

### 2.2. The Chemical Bonding Analysis on Au_10_ Cluster

#### 2.2.1. AdNDP Analysis

**Figure 2:**
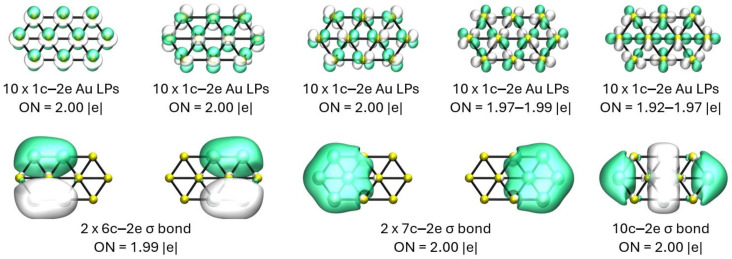
(Color online) Results of the AdNDP analysis for the lowest energy structure of the Au_10_ system. Each green and white isosurface represents the N-center orbital at each vertex of the cluster, respectively.

To shed light in the bonding of the Au_10_ cluster, we carry out the chemical bonding analysis on the 2D elongated hexagon structure depicted in Figure 1 (1) and also on the 3D capped trigonal prism displayed in Figure 1 (6). The results of the AdNDP analysis for the lowest energy structure, displayed in Figure 1 (1), are shown in Figure 2. This analysis reveals the presence of five lone pairs (LPs) on each of the Au atoms with ON values very close to the optimum value of 2 |e|, which indicates that the Au d orbitals do not participate in the bonding of this system. The bonding pattern is completed by five delocalized sigma bond elements as follows: two 6c–2e bonds involving the hexagonal ends, two 7c–2e bonds involving the hexagonal ends plus the Au atom in the center of each hexagon, and, finally, a 10c–2e bond delocalized over the entire structure.

**Figure 3:**
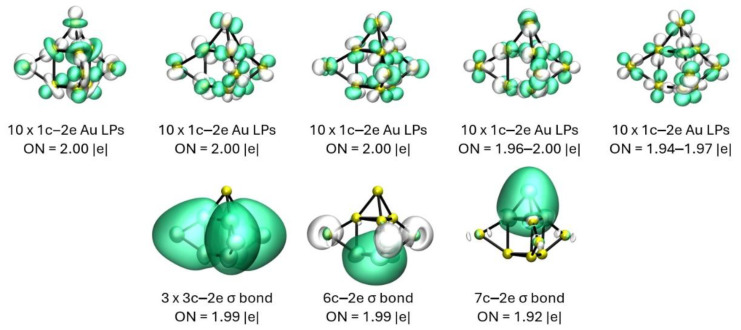
Shows the results of the AdNDP analysis for the 3D capped trigonal prism-shaped isomer displayed in Figure 1 (6). Each green and white isosurface represents the N-center orbital at each vertex of the cluster, respectively.

The analysis f or the 3D structure reveals the five LPs on each Au atom, while the remaining bonding pattern consists of three 3c–2e bonds, each of them involving the Au atom that is capping the vertical axis of the prism and the two atoms of the same axis, and two delocalized sigma bonds: one of 6c–2e and the other of 7c–2e, whose main contribution comes from the lower and upper triangular faces of the prism, respectively. Figure 3 displays the results of the AdNDP analysis for the 3D low-energy structure of the Au_10_ system.

#### 2.2.2. QTAIM Analysis

The QTAIM parameters computed at the BCP provide information about the nature of the interaction between the two Au atoms [64].

**Figure 4:**
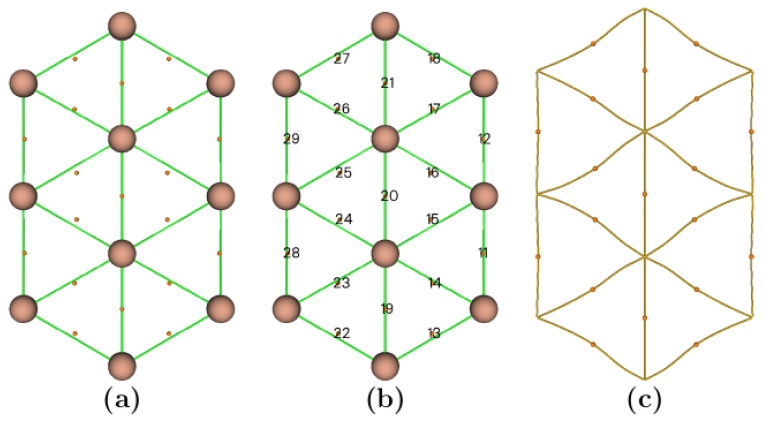
(Color online) Molecular graphs for the lowest energy structure Au_10_ cluster. (**a**) The BCPs are depicted in red-yellow color small spheres; (**b**) Index of the BCPs (numbers); (**c**) Bond paths (BPs) are the lines of maximum density that connects a pair of nuclear critical points. The BPs are used as an indicator of chemical bonding [76] between a pair of Au atoms. The atoms are depicted as red-brown spheres.

The total number of critical points of each type we found for the lowest energy structure of the Au_10_ cluster is 10 NCP, 19 BCP, and 10 ring critical points. Figure 4a displays the BCP points, Figure 4b displays the index of BCP, and Figure 4c displays the BP paths. We found 39 CP points, which fulfill the Poincaré–Hopf relationship. According to our computed CP (Figure 4a), 19 BCPs indicate chemical bonds among Au–Au atoms. Additionally, Figure 4c shows the BP indicative of chemical bonding. To shed light on the chemical bonding Au–Au, in each BCP, we evaluated these QTAIM parameters: the electronic density (ρ), the Laplacian of electron density (∇^2^ρ), and the energy density H(r), that are displayed in Table 1. Analyzing the data of Table 1, the Au–Au average bond distance between Au–Au atoms is 2.70 Å, which agrees with the bond distance reported for gold nanoclusters, which are in the range of 2.6–2.84 Å [63]. From Table 1, the Laplacian of electron density is positive for all BCPs; the positive value of the Laplacian at the BCP points indicates a depletion of the density [63]. Thus, according to the values displayed in Table 1, the Au–Au bond is non-covalent, which agrees with previous work [63]. Similar weak non-covalent Pd–Pd interactions have been reported in the metallic (Pd–Pd) and heterobimetallic (Pd–Ir) clusters [75]. The positive Laplacian of electron density and negative energy density values indicate a closed-shell Au–Au interaction with a weak or partially covalent character.

### 2.3. Energetics

To shed light on the proper energy hierarchy of the Au_10_ isomers and compare them with DFT energies. Single-point energies of each isomer were computed employing the method of DLPNO-CCSD(T) compared with those obtained at the DFT level of theory. Previous studies pointed out that, employing different methods to compute energies yields different results [34], particularly in small gold clusters [28].

Table 2, in the first row, displays the number of isomers following the label cluster given in Figure 1. The second row of Table 2 displays the relative DFT Gibbs free energy computed at PBE0-D3BJ and ZORA SARC-ZORA-TZVP SARC/J level of theory and at 100 K. The third row of Table 2 displays single-point energy computed at the DLPNO-CCSD(T) level of theory plus ZPE energy. This follows the energetic ordering of the second row of Table 2 at the DFT level of theory. The second row of Table 2 indicates that the putative global minimum is the planar elongated hexagon structure depicted in Figure 1 (1), according to single-point energy calculation at the DLPNO-CCSD(T) level of theory, as displayed in the third row of Table 2. This isomer lies at 4.26 kcal/mol above the putative lowest energy structure. This structure is composed of a trigonal prism as the main central structure that is edge-capped by four atoms, as depicted in Figure 1 (6). Moreover, it is labeled as isomer number six in Table 2. In summary, Table 2 indicates that the lowest energy structure is the 2D elongated hexagon structure computed at the DFT level of theory. In contrast, at the DLPNO-CCSD(T) + ZPE level of theory, the 3D capped trigonal prism is the lowest energy structure. According to DLPNO-CCSD(T) SP energies, the second isomer, labeled number 4 in Table 2, is located at 0.03 kcal/mol above the putative global minimum. The next isomer, labeled 6 in Table 2, is the putative global minimum at the DLPNO-CCSD(T) level of theory. The computed thermal population for those three isomers indicates that they contribute 0.59, 0.49, and 0.41%, respectively. The following isomers, labeled from 7 to 12 in Table 2, exhibit a continuous increase in energy without exchanges and in agreement with DFT energy ordering.

## 3. Theoretical Methods and Computational Details

### 3.1. Lowest Energy Structure Search

The search for the lowest energy structure and the low-energy structures is a complicated task due to several factors: (a) the exploration of the potential energy surface should be systematic and unbiased [77]; (b) the number of possible combinations of atomic arrangements grows exponentially with the number of atoms leading to a combinatorial explosion problem [44,78]; (c) computation of the total energy requires quantum mechanical DLPNO-CCSD(T) level of theory to achieve a realistic value of energy; (d) sampling a large region of the configuration space [79]. Several methods and theoretical studies have been developed to search for the lowest energy structures. The design and use of algorithms, like simulated annealing [80,81,82,83,84,85], kick method [86,87], gradient embedded genetic algorithm (GEGA) [88,89,90], basin hopping [91,92], among others [93], helped to explore the potential energy surface. Previous work employed genetic algorithms [94,95,96,97,98] and kick methodology [97,99,100,101,102,103,104,105,106] coupled with density functional theory with the aim of exploring the potential energy surface. In this study, our computational procedure employs a genetic algorithm coupled to the Orca code and implemented in the GALGOSON code [34,43,107]. The methodology employs a three-step search strategy, the optimization in this first stage was at the PBE0 [68] and LANL2DZ [108] level of theory. In previous studies, PBE0 was employed to compute the density of states, which yielded good agreement with the observed spectrum for anion Au_19_ [109]. The LANL2DZ basis set is employed for transition metals due to its low computational cost [34,110], and as the second step, structures lying into ten kcal/mol found in the previous step were optimized employing the PBE0 functional and utilizing the SARC-ZORA-TZVP basis set [69], in conjunction with the auxiliary basis SARC/J [70] and using the AutoAux generation procedure [71]. The dispersion is taken into account by employing the atom-pairwise dispersion correction with the Becke–Johnson damping scheme (D3BJ) [67]. The relativistic effects are considered through SARC-ZORA-TZVP. Additionally, we ensure that each isomer is a proper local low-energy structure through the vibrational mode of each isomer, confirming that the lowest vibrational mode of each isomer is positive. As the third step, the single-point energy calculations at the DLPNO-CCSD(T) level were performed using the version 5.0 of the ORCA quantum chemistry program suite with TightPNO settings [72].

### 3.2. Thermochemistry Properties

The partition function describes all statistical thermodynamic properties of an ensemble of atoms at thermodynamic equilibrium. Here, we computed the thermodynamic properties employing the partition function Q displayed in Equation (1).

(1) $$Q\left(T\right)=\sum_{i}g_{i}e^{-{\Delta E}_{i}/k_{B}T}$$

In Equation (1), $g_{i}$ is the degeneracy factor, $k_{B}$ is the Boltzmann constant, T is the absolute temperature, and ${\Delta E}_{i}$ is the total electronic energy of a cluster [111,112]. Due to the coupling of the internal modes, the computation of Equation (1) could be complicated [43]. The starting point for computing the thermochemical data is the partition function given in Equation (1), and it can be decomposed as contributions from ${\Delta E}_{i}$ rotational, translation, vibration, and electronic. Therefore, the total partition function of a molecule in its ground state is partition functions, so we can re-write Equation (1) as a product of partial partition functions [43,111,113] as given in Equation (2).

(2) $$q=q_{trans}q_{rot}q_{vib}q_{elec}$$

All Equations displayed in Table 3 are computed at a standard pressure of 1 atm and finite temperature, employing ideal gas statistical mechanics. The equations are equivalent to those given in Ref. [111] and any standard text of thermodynamics [112,113]. In this study, the $q_{trans}$ is computed at a finite temperature and is employed to compute the translational entropy. The rotational contribution depends on moments of inertia and rotational symmetry number [33]; the so-called rigid rotor harmonic oscillator approximation is employed to separate the rotational and vibrational modes [43]. Previous works computed the difference in the rotational entropy computed with and without the symmetries [33]. To address the failure of the harmonic oscillator approximation at low frequencies, entropic contributions to the free energies are calculated by default using Grimme’s Quasi-RRHO approach [57]. The electronic partition function, $q_{elec}$, is given by $q_{elec}$ = ω0. The contribution to the total energy of a molecule at a finite temperature is a sum of electronic, translational, vibrational, and rotational energies. We employ equations 3–6 to compute the internal energy (U), enthalpy (H), and Gibbs energy (G) of the Au_10_ cluster at finite temperature. The rotational contribution to the entropy is calculated using the expressions given by Herzberg [114].

(3) $$U_{0}=\varepsilon_{0}+ZPE$$

(4) $$U_{T}=U_{0}+\left(E_{Rot}+E_{Trans}+E_{Vib}+E_{elect}\right)$$

(5) $$H=U_{T}+nRT$$

(6) ΔG=ΔH−ΔST

In the Equations above, ZPE is the zero-point energy correction, $\varepsilon_{0}$ is the electronic energy, and $E_{Rot}+E_{Trans}+E_{Vib}+E_{elect}$ are the contributions to energy due to translation, rotation, electronic, and vibration as a function of temperature, respectively. In Equation (5), *R* is the ideal gas constant, *n* is the amount of substance, and T is the absolute temperature.

### 3.3. Thermal Population

In a molecule, the measured properties represent the statistical averages of the ensemble of geometric conformations or isomers available to the cluster [115] in an ensemble at thermal equilibrium. We computed the thermal population or probabilities of occurrence [34,56,79,111,116,117,118] employing Equation (7).

(7) $$P\left(T\right)=\frac{e^{-{\beta \Delta G}^{K}}}{\sum e^{-{\beta \Delta G}^{K}}}$$

where $\beta =1/k_{B}T$, $k_{B}$ is the Boltzmann constant, T is the temperature, and ${\Delta G}^{k}$ is the Gibbs free energy of the *k*th isomer. Equation (7) is restricted so that the sum of all the thermal population at fixed temperature T, $P_{i}\left(T\right)$, must be equal to 1, according to Equation (8).

(8) $$\sum_{i=1}^{n}P\left(T\right)=1$$

In this study, ${IR}_{Bolt}$ is the Boltzmann-weighted spectrum at temperature T which is given by Equation (9).

(9) $${IR}_{Bolt}=\sum_{i=1}^{n}\left({IR}_{i}\right)xP_{\left(i\right)}\left(T\right)$$

where *n* is the total number of clusters in the ensemble, and ${IR}_{i}$ is the IR of the *i*th isomer at temperature T = 0, and $P_{i}\left(T\right)$ is the probability of the *i*th isomer given by Equation (7).

### 3.4. The Chemical Bonding

The chemical bonding analysis was performed employing the adaptive natural density partitioning method [119], which can be seen as an extension of the natural bond orbital (NBO) [120,121,122] analysis in which both Lewis bonding (localized 1c–2e lone-pairs and 2c–2e bonds) and delocalized bonding elements are recovered from the analysis of the first-order reduced density matrix in the basis of the natural atomic orbitals (NAOs). The procedure consists of obtaining the transformation matrix between the basis set of atomic orbitals, the basis set of NAOs, and the first-order-reduced density matrix in the basis of these NAOs. Then, in the AdNDP analysis, a block diagonalization of this density matrix is performed, obtaining the corresponding eigenvectors (bonding elements) and their eigenvalues (occupation numbers, ON), which are selected if the ON is close enough to the threshold of 2.00 |e|. The transformation and first-order reduced density matrices were obtained at the PBE0/LANL2DZ level using the NBO7 program [123] interfaced with the Gaussian package. The AdNDP analysis was carried out with the freely MultiWFN program v3.8 [124].

Additionally, we used the quantum theory of atoms in molecules (QTAIM) [125,126] for determining the chemical bonding at the lowest energy structure of Au_10_ cluster employing the freely MultiWFN program v3.8 [124]. The electron density topology is based on the critical point (CP) concept. The CP is defined as the point where the first derivative of the electron density vanishes. The bond critical point (BCP) is found between two nuclear critical points (NCPs) and indicates the presence of a chemical bond.

## 4. Thermal Population

**Figure 5:**
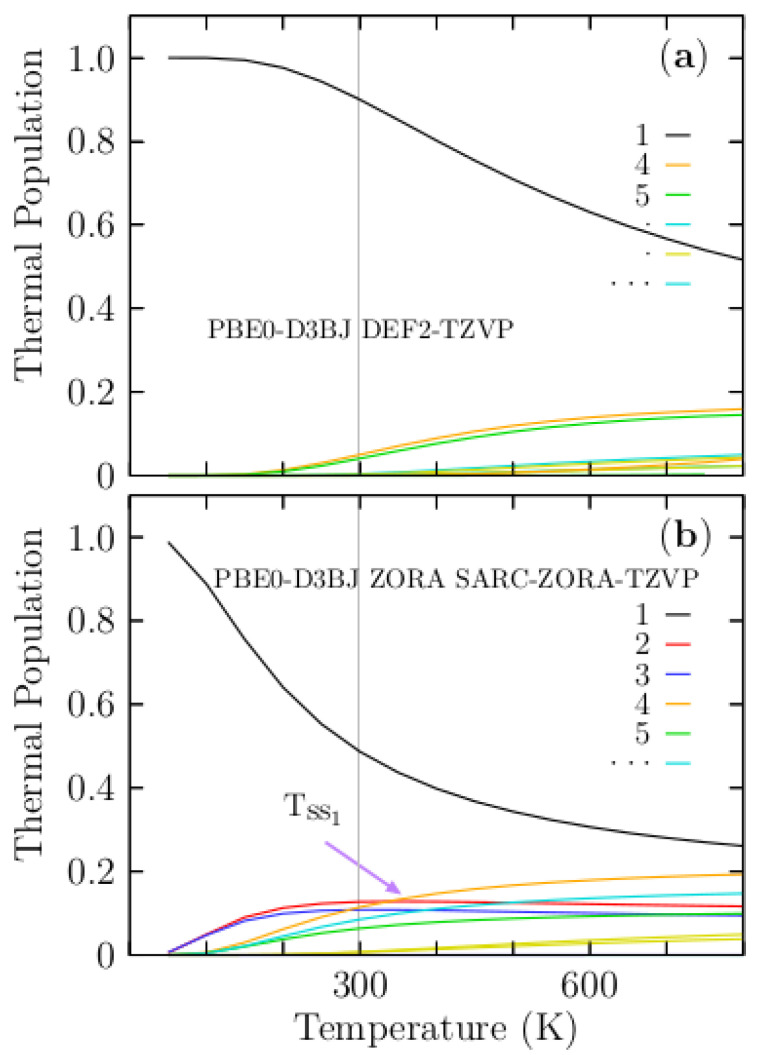
(Color online) The thermal population for all Au_10_ isomers at temperatures ranging from 50 to 800 K. For an easy comparison, in panel (**a**), we show the thermal population without relativistic effects. In panel (**b**), we show the thermal population where relativistic effects are taken through zero-order regular approximation (ZORA) [131].

In this section, based on the computed free-energy differences among Au_10_ isomers, we calculated the thermal population [34,56,79,111,116,117,118] or Boltzmann distribution at finite temperature and as standard pressure, as given in Equation (7). The observed molecular properties are statistical averages over a collection of molecules. Consequently, the statistical averages are crucial for the experimental measurement of thermodynamic quantities [127]. Distinguishing isomers based on their energy differences is essential for determining the solid–solid transition point within the thermal population. The thermal population is related to several solid–solid transition points if free energy differences between the putative lowest energy structure and the first low-energy isomer are small. Conversely, if those free energy differences are considerably greater, the thermal population has no solid–solid transition points. Additionally, free-energy differences are related to the fundamental chemical quantities such as binding constants, solubilities, partition coefficients, and adsorption coefficients [128,129,130]. Figure 5a, displays the thermal population for each particular Au_10_ isomer for temperatures ranging from 50 to 800 K. We computed that the thermal population at the PBE0 Def2-TZVP def2/J RIJCOSX level of theory takes into account the D3BJ dispersion correction method. In Figure 5a, the thermal population of the lowest energy planar elongated hexagon configuration is depicted by a black solid line, and at temperatures ranging from 50 to 150 K, the thermal population is constant. At 150 K, it began to decay slowly exponentially up to room temperature, where it achieved 90% probability. Above room temperature, the thermal population decays almost linearly, achieving 50% at 800 K, as displayed in the upper panel of Figure 5a. The analysis of these results led to three interesting conclusions in temperatures ranging from 50 to 800 K. (1) There are no solid–solid transformation points, (2) the planar elongated hexagon configuration strongly dominates, and (3) all molecular properties of the Au_10_ cluster are attributed to planar-elongated hexagon configuration. The thermal population of the isomer, an edge-capped trigonal prism displayed in Figure 1 (4), is depicted by a red solid line in Figure 5a. At a temperature of 150 K, it starts to increase, achieving 5% probability at room temperature, and at a temperature of 800 K, it achieves almost 15% of probability. This low-energy structure does not contribute to the molecular properties. Figure 5b, displays the thermal population of Au_10_ computed in the temperature range of 50 to 800 K and at PBE0 ZORA SARC-ZORA-TZVP UseSym SARC/J AUTOAUX level of theory, taking into account the D3BJ dispersion correction method and relativistic effects through ZORA correction. The planar elongated hexagon structure strongly dominates in the temperatures range of 50–800 K. At 50 K, the probability of a planar elongated hexagon configuration is 98% and decays exponentially fast to achieve 58% probability at room temperature; above room temperature, the probability decays exponentially until it achieves 30% at 800 K. The solid–solid transformation point (T_ss1_) shown in Figure 5b, is located at 350 K, and occurs between isomers two and four located higher in energy above the putative global minimum, and other Tss points with lower probabilities less than 10%, may occurs around room temperature point. Notice that, in Figure 5a,b, the planar elongated hexagon configuration strongly dominates the thermal population. Moreover, relativistic effects do not significantly change the thermal population. At 50 K, the probability achieves almost 100%. Therefore, based on our calculations, the molecular properties of Au_10_ are mainly due to the putative global minimum energy structure, planar elongated hexagon configuration, in a temperature range of 50–800 K.

## 5. Infrared Spectra (IR) of Au_10_ Cluster

**Figure 6:**
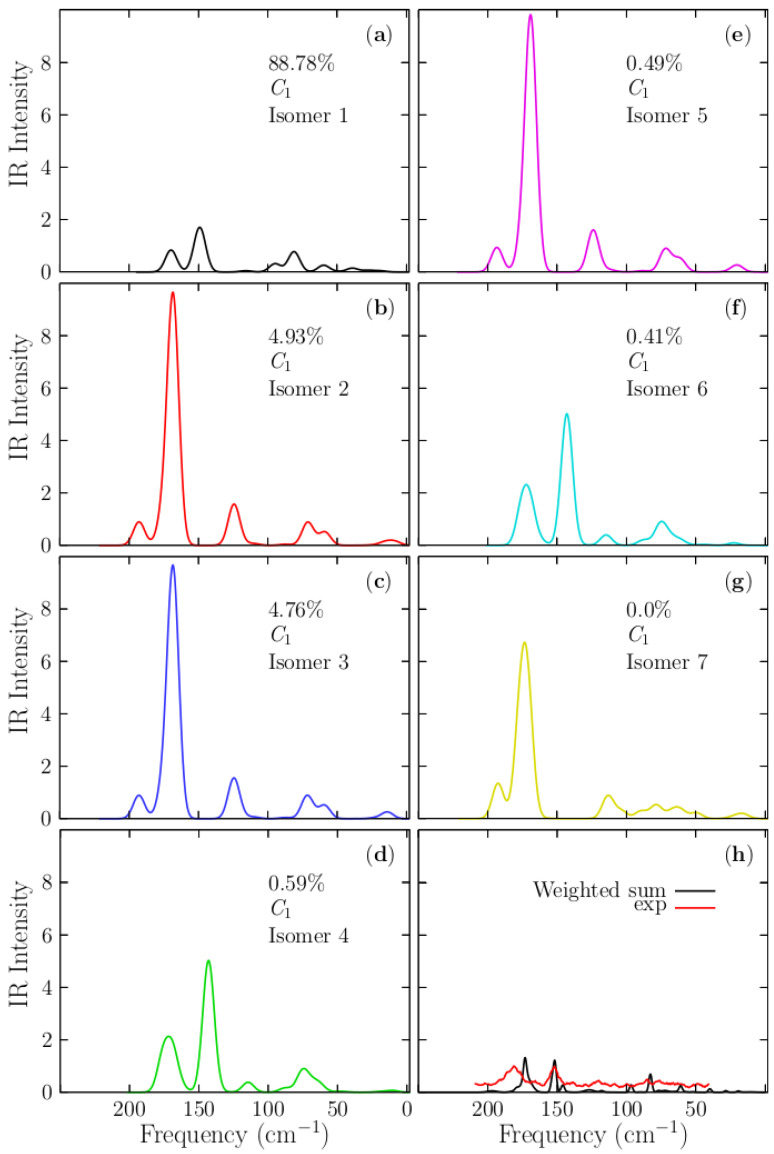
(Color online) At 0 K, the IR spectra of Au_10_ isomers were computed in the frequency range of 50–250 cm^−1^. Panel (**a**–**g**) display the IR spectra of each low–energy isomer computed with Version 5.0 of the ORCA quantum chemistry program suite. Panel (**h**) compares the IR experimental, and the Boltzmann spectra computed at 100 K and depicted as a black solid line. The red solid line represents the experimental IR spectrum from references [11,41].

IR spectra are usually used to identify functional groups and bond chemical information; however, from the experimental point of view. Identifying which IR bands correspond to vibrational molecular modes can be complex and demands ab initio calculations. In these computations, temperature is often not considered, and discrepancies between experimental and theoretical IR spectra can arise from finite temperature and anharmonic effects. In this study, each spectrum of each isomer was computed using DFT, as it is implemented in the Gaussian 09 code. In Figure 6a–g, present the IR spectra for the presumed global minimum and the six lowest energy configurations, found within the relative energy range of 0–2.49 kcal/mol. The Figure 6h compares the computed Boltzmann spectrum of the Au_10_ cluster with the observed IR spectrum [11,41]. The Boltzmann weighted spectrum at 100 K is depicted in a black solid line. The red solid line represents the experimental infrared spectrum of Au_10_ taken from references [11,41]. The experimental spectrum in Figure 6h, shows two peaks located at 150 cm^−1^ and 180 cm^−1^, respectively. The spectrum is almost flat in the range of 50–150 cm^−1^. Interestingly, the computed IR Boltzmann spectrum is in good agreement with the experimental studies of Goldsmith et al. [11,41]. The peak of the IR Boltzmann spectrum, depicted with a black solid line located at 150 cm^−1^, shows good agreement with the experimental IR spectrum, as shown in Figure 6h.

However, the intensities of those peaks are similar; the Boltzmann spectrum is narrower than the experimental one. The vibrational mode of the peak is located at 150 cm^−1^, consisting of stretching and oscillating the two central atoms along the larger axis of the cluster and within the plane. Whereas the peak located at 180 cm^−1^ consists of the stretching and oscillation of the two central atoms along the shorter axis of the cluster and within the plane. Interestingly, 88.7% of the Boltzmann IR spectrum, depicted in the black solid line and displayed in Figure 6h, comes from the IR spectrum of the putative global minimum. The other 11.3% of the Boltzmann IR spectrum is composed of contributions from the six low-energy structures.

## 6. Conclusions

In summary, our study systematically explored the potential and free-energy surfaces of the Au_10_ cluster. We achieved this using an efficient genetic algorithm written in Python 2.7 coupled with density functional theory as implemented in the version 5.0 of the ORCA quantum chemistry program suite. The study highlights the findings of elucidate the lowest energy structure of the Au_10_ cluster, considering the relativistic corrections, dispersion, and temperature. We consider relativistic effects through the ZORA approximation, the dispersion through the D3BJ Grimme methodology, and the effects of temperature are included through nanothermodynamics.

The computed thermal population indicates that the planar elongated hexagon configuration strongly dominates in the temperature range from 0 to 800 K, regardless of whether ZORA is considered or not. Overall, relativistic corrections and temperature effects do not result in a transition from the 2D planar to a 3D Au_10_ structure. We also compared the energetic ordering of the low-energy structures computed at the DFT level of theory versus the single-point energies employing the DLPNO-CCSD(T) level of theory, which indicated an interchange in the energetic ordering of the isomers, determining a 3D structure as the lowest energy structure.

We computed the IR spectrum at 100 K employing the Boltzmann methodology. This agrees with experimental studies conducted by B. R. Goldsmith et al. The study highlights that the 2D elongated hexagon configuration is the putative global minimum in the temperature range from 0 to 800 K; consequently, the transition from 2D to 3D does not occur at the Au_10_ cluster.

The 2D elongated hexagon structure’s AdNDP analysis indicates that the bonding of the cluster is solely due to the electrons of the 6 s orbital, with no participation of the Au-d orbitals. The QTAIM analysis indicates that the positive Laplacian of electron density and negative energy density values (on BCP) indicate a closed-shell Au–Au interaction with a weak covalent character. For the capped trigonal prism-shaped isomer, the AdNDP analysis for this structure reveals the five LPs on each Au atom, while the remaining bonding pattern consists of three 3c–2e bonds. A forthcoming project involves computing the optical spectra of Au_10_ clusters using the Boltzmann scheme.

## Figures and Tables

**Table 1:** (1) Indexes of BCP as they are displayed in Figure 4b. (2) Electronic density (ρ). (3) Laplacian of electron density (∇^2^ρ). (4) Energy density H(r). (5) Bond distances.

Indexes BCP	ρ	∇^2^ρ	H(r)	Bond Distance
14	0.5748	0.1145	−0.1810	2.6892
19	0.7227	0.1291	−0.2436	2.6770
23	0.5748	0.1145	−0.1810	2.6892
24	0.5358	0.1100	−0.1478	2.7173
20	0.7130	0.8501	−0.2287	2.7304
15	0.5358	0.1100	−0.1478	2.7173

**Table 2:** The first row of the table displays the number of isomers following the ordering in Figure 1. The second row shows the relative DFT Gibbs free energy computed at the PBE0-D3BJ and ZORA SARC-ZORA-TZVP SARC/J level of theory, and at 100 K. The third row shows the relative single point energy (SPE) computed employing DLPNO-CCSD(T) level of theory, with the TightPNO setting and ZPE energy correction based on DFT.

Energy DFT versus DLPNO-CCSD(T) SPE Energy		
No. of Isomer (Figure 1)	DFT Energy (kcal/mol)	DLPNO-CCSD(T) SPE Energy (kcal/mol)
1	0.0	4.26
2	0.57	8.44
3	0.58	9.34
4	0.99	0.03
5	1.03	0.53
6	1.06	0.0
7	2.43	3.18
8	2.47	3.73
9	3.51	3.92
10	5.89	4.56
11	7.27	7.11
12	8.59	15.08

**Table 3:** The contribution of electronic, translational, vibrational, and rotational partition functions to Equation (1).

Contribution	Partition Function
Translational	$q_{\mathrm{trans}}={\left(\frac{2\pi mk_{B}T}{h^{2}}\right)}^{\frac{3}{2}}\frac{k_{B}T}{P}$
Rotational linear	$q_{\mathrm{rot}}^{l}=\frac{1}{\sigma_{r}}\left(\frac{T}{\Theta_{r}}\right)$
Rotational nonlinear	$q_{rot}^{nl}={\left(\frac{\pi }{\Theta_{A}\Theta_{B}\Theta_{C}}\right)}^{1/2}\left(\frac{T^{3/2}}{\sigma_{r}}\right),\Theta_{i}=\frac{\hbar^{2}}{2I_{i}k_{B}},i=A,B,C$
Vibrational	$q_{\mathrm{vib}}=\prod_{i=1}^{n_{v}}\frac{e^{-\Theta_{\mathrm{vib},i}/2T}}{1-e^{-\Theta_{\mathrm{vib},i}/T}},\Theta_{\mathrm{vib},i}=\frac{h\nu_{i}}{k_{B}}$
Electronic	$q_{e}=\omega_{0}$

## Data Availability

Data are contained within the article and Supplementary Materials.

## Supplementary Materials

The following supporting information can be downloaded at: https://www.mdpi.com/article/10.3390/molecules29143374/s1, The xyz atomic coordinates of the optimized Au_10_ cluster.

## Abbreviations

DFT	Density Functional Theory
DLPNO-CCSD(T)	Domain-based Local Pair Natural Orbital Coupled-Cluster Theory
ZPE	Zero-Point Energy
IR	Vibrational Infrared Spectrum
BOFA	Boltzmann-Optics-Full-Ader code (Module Nanothermodynamics)
GALGOSON	Global Genetic Algorithm
