# Supplementary material for: Exploration of Free Energy Surface of the Au10 Nanocluster at Finite Temperature
Source: Molecules. 2024 Jul 18;29(14):3374. doi: 10.3390/molecules29143374 (PMC11279810; doi:10.3390/molecules29143374)
Supplement: Supplementary file 1 [file molecules-29-03374-s001.zip › molecules-3052005-supplementary.pdf]

molecules

## Supplementary Material

# Exploration of Free Energy Surface of the $Au_{10}$ Nanocluster at Finite Temperature

**Francisco Eduardo Rojas-González [1], César Castillo-Quevedo [2], Peter Ludwig Rodríguez-Kessler [3], José Oscar Carlos Jimenez-Halla [4], Alejandro Vásquez-Espinal [5], Rajagopal Dashinamoorthy Eithiraj [6], Manuel Cortez-Valadez [7] and José Luis Cabellos [8,*]**

1 Departamento de Física, Edificio 3F, Universidad de Sonora, Hermosillo 83000, Sonora, Mexico; francisco.rojas@unison.mx
2 Departamento de Fundamentos del Conocimiento, Centro Universitario del Norte, Universidad de Guadalajara, Carretera Federal No. 23, km. 191, Colotlán 46200, Jalisco, Mexico; castillo.quevedo@cunorte.udg.mx
3 Centro de Investigaciones en Óptica, A.C. (CIO) Lomas del Bosque 115, León 37150, Guanajuato, Mexico; plkessler@cio.mx
4 Departamento de Química, División de Ciencias Exactas y Naturales, Universidad de Guanajuato, Noria Alta s/n, Guanajuato 36050, Guanajuato, Mexico; jjimenez@ugto.mx
5 Química y Farmacia, Facultad de Ciencias de la Salud, Universidad Arturo Prat. Casilla 121, Iquique 1100000, Chile; alvasquez@unap.cl
6 Department of Physics, School of Advanced Sciences, Vellore Institute of Technology (VIT), Chennai 600 127, Tamil Nadu, India; eithiraj.rd@vit.ac.in
7 CONAHCYT-Departamento de Investigación en Física, Universidad de Sonora, Apdo. Postal 5-88, Hermosillo 83190, Sonora, Mexico; manuelcortez@live.com
8 Coordinación de Investigación y Desarrollo Tecnológico, Universidad Politécnica de Tapachula, Carretera Tapachula a Puerto Madero km. 24, Tapachula 30830, Chiapas, Mexico
* Correspondence: jose.cabellos@uptapachula.edu.mx

XYZ atomic coordinates $Au_{10}$ cluster. (Figure 1)

10
1

| | | | |
|---|---|---|---|
| Au | -2.68205400000 | 2.3448280000000 | 0.000000000000 |
| Au | 4.042240000000 | 0.000006000000 | 0.000000000000 |
| Au | -2.682080000000 | -2.344830000000 | 0.000000000000 |
| Au | 2.682077000000 | -2.344826000000 | 0.000000000000 |
| Au | 0.000001000000 | 2.349466000000 | 0.000000000000 |
| Au | -4.042241000000 | 0.000005000000 | 0.000000000000 |
| Au | -1.365241000000 | -0.000002000000 | 0.000000000000 |
| Au | 0.000000000000 | -2.349474000000 | 0.000000000000 |
| Au | 2.682057000000 | 2.344828000000 | 0.000000000000 |
| Au | 1.365240000000 | -0.000001000000 | 0.000000000000 |

10
2

Au -0.158551479028 -1.377683471946 1.430365120251
Au 1.968201407406 0.242901032195 -1.384689619650
Au -0.489141801819 1.298663212660 -1.431555279589
Au -0.158551479028 -1.377683471946 -1.430365120251
Au -1.919521543105 -2.905324309932 0.000000000000
Au 4.215819234413 0.504897639360 0.000000000000
Au 1.968201407406 0.242901032195 1.384689619650
Au -2.372340033350 -0.287671855165 0.000000000000
Au -0.489141801819 1.298663212660 1.431555279589
Au -2.564973911076 2.360336979919 0.000000000000
10
3
Au -2.632615644853 2.813888862326 0.000000000000
Au 1.301587909103 -0.015845065641 1.396872200483
Au 0.888409292315 -1.745124641874 -3.317360739158
Au 0.888409292315 -1.745124641874 3.317360739158
Au 1.301587909103 -0.015845065641 -1.396872200483
Au -0.883385047015 -1.651941554390 -1.337620936187
Au 2.499824979686 1.935874480286 0.000000000000
Au -2.298073279590 0.178975070548 0.000000000000
Au -0.182360364049 1.897084110647 0.000000000000
Au -0.883385047015 -1.651941554390 1.337620936187
10
4
Au -1.614609452657 3.087404536894 0.000000000000
Au 4.142163475195 -0.951814009006 0.000000000000
Au -0.014082906437 1.385613606259 1.426977000000
Au 1.936714742145 -0.445032296195 -1.384660500000
Au -0.617522136690 -1.240476191754 -1.426972000000
Au 1.936714742145 -0.445032296195 1.384660500000
Au -0.617522136690 -1.240476191754 1.426972000000
Au -2.800368368724 -2.072901244548 0.000000000000
Au -2.337405051850 0.537100480041 0.000000000000
Au -0.014082906437 1.385613606259 -1.426977000000
10
5
Au -2.265027727227 2.647391141092 0.000000000000
Au 4.250113899997 0.000005528531 0.000000000000
Au -0.324035454809 1.347265533639 1.426977000000
Au 1.987188400000 0.000001573293 -1.384660500000
Au -0.324030745196 -1.347263466357 -1.426972000000
Au 1.987188400000 0.000001573293 1.384660500000
Au -0.324030745196 -1.347263466357 1.426972000000

Au -2.265010472770 -2.647396858886 0.000000000000
Au -2.398320099991 -0.000007091886 0.000000000000
Au -0.324035454809 1.347265533639 -1.426977000000

10
6
Au 1.879103127390 0.573325153140 3.317579991681
Au -1.492573698448 -1.190022305258 0.000000000000
Au 1.879103127390 0.573325153140 -3.317579991681
Au -3.855104548176 -0.066425760130 0.000000000000
Au 0.909315838033 -0.920203241795 -1.396542739494
Au 0.573900895375 1.782853893140 -1.337418368719
Au 0.350631007239 -3.143016496452 0.000000000000
Au 0.573900895375 1.782853893140 1.337418368719
Au 0.909315838033 -0.920203241795 1.396542739494
Au -1.727592482213 1.527512952870 0.000000000000
10
7
Au -0.678196811155 -3.101858413470 -1.703639727749
Au -1.033799790934 1.644348983000 -0.358931735036
Au 0.678196811155 3.101858413470 -1.703639727749
Au -1.634933616546 -1.080145601431 -0.341750675216
Au 1.033799790934 -1.644348983000 -0.358931735036
Au -3.460477639104 0.758348552810 0.376157337545
Au 1.264718937425 -0.279492935091 2.028164800456
Au -1.264718937425 0.279492935091 2.028164800456
Au 1.634933616546 1.080145601431 -0.341750675216
Au 3.460477639104 -0.758348552810 0.376157337545
10
8
Au -3.375143021574 1.313427003023 0.000000000000
Au 0.037678001367 -0.358562921530 3.540901500000
Au 1.352897870516 0.503102497108 -1.362980500000
Au -1.427920596661 0.210912742436 -1.362982500000
Au -1.427920596661 0.210912742436 1.362982500000
Au 0.037678001367 -0.358562921530 -3.540901500000
Au 1.352897870516 0.503102497108 1.362980500000
Au 0.210703949879 -2.005298775499 1.295900000000
Au 0.210703949879 -2.005298775499 -1.295900000000
Au 3.028424571372 1.986265911947 0.000000000000
10
9

| | | | |
|---|---|---|---|
| Au | 3.305011522266 | 2.598387039357 | 0.000000000000 |
| Au | -3.768961682768 | -1.330630595974 | 0.000000000000 |
| Au | -3.768949317260 | 1.330632404065 | 0.000000000000 |
| Au | 3.293622999968 | 0.000017010957 | 0.000000000000 |
| Au | -1.430038626934 | 2.658229234941 | 0.000000000000 |
| Au | 0.936020458200 | 1.420707133491 | 0.000000000000 |
| Au | -1.430043373021 | -2.658250765087 | 0.000000000000 |
| Au | -1.377757999993 | -0.000004634732 | 0.000000000000 |
| Au | 0.936027541826 | -1.420714866501 | 0.000000000000 |
| Au | 3.305068477716 | -2.598371960517 | 0.000000000000 |
| 10 | | | |
| 10 | | | |
| Au | -1.835641767160 | 3.858636862885 | 0.000000000000 |
| Au | -2.165563659282 | 1.262795865394 | 0.000000000000 |
| Au | 0.337482355386 | 2.338002379900 | 0.000000000000 |
| Au | -3.286195029006 | -1.108819950227 | 0.000000000000 |
| Au | -0.774652962915 | -0.955645260191 | 0.000000000000 |
| Au | 1.577971259808 | -1.987453608004 | 2.584229365580 |
| Au | 1.547115090397 | -2.138719300978 | 0.000000000000 |
| Au | 1.577971259808 | -1.987453608004 | -2.584229365580 |
| Au | 1.510756726482 | 0.359328309612 | -1.353420571141 |
| Au | 1.510756726482 | 0.359328309612 | 1.353420571141 |
| 10 | | | |
| 11 | | | |
| Au | -4.047789431288 | -1.659351591093 | 0.000000000000 |
| Au | -1.500105098038 | -2.167971002576 | 0.000000000000 |
| Au | -2.444149495626 | 0.449410942841 | 0.000000000000 |
| Au | -2.926956051183 | 3.025914016511 | 0.000000000000 |
| Au | -0.388216518622 | 2.273373605718 | 0.000000000000 |
| Au | 2.185511645769 | 1.656887769503 | 0.000000000000 |
| Au | 4.737827124278 | 0.781534426567 | 0.000000000000 |
| Au | 2.949643548082 | -1.086891220118 | 0.000000000000 |
| Au | 1.055174578264 | -2.970705001317 | 0.000000000000 |
| Au | 0.379059698362 | -0.302201946037 | 0.000000000000 |
| 10 | | | |
| 12 | | | |
| Au | 2.904174701623 | -0.055923647213 | -1.391439720849 |
| Au | 0.706859601774 | -1.653255584535 | 1.361668124226 |
| Au | 2.904174701623 | -0.055923647213 | 1.391439720849 |
| Au | 0.476828092105 | 1.041186412264 | -1.389535532148 |
| Au | -0.569074969523 | 3.112631597873 | 0.000000000000 |
| Au | 0.476828092105 | 1.041186412264 | 1.389535532148 |
| Au | 0.706859601774 | -1.653255584535 | -1.361668124226 |

```
Au   -1.605779569789     -1.855632718861      0.000000000000
Au   -4.037704923063     -0.762997025475      0.000000000000
Au   -1.963165328628      0.841983785430      0.000000000000
```

## XYZ Atomic coordinates (exploration).

```
10
0.000000000         1
Au   0.002985000000   1.552645000000  -1.715570000000
Au   1.416831000000  -0.819238000000   1.023103000000
Au   1.343138000000  -0.778907000000  -1.715570000000
Au   0.000000000000   0.000000000000   3.177129000000
Au  -1.346123000000  -0.773738000000  -1.715570000000
Au  -1.417896000000  -0.817393000000   1.023103000000
Au   0.000000000000   3.868966000000  -0.366576000000
Au  -3.350623000000  -1.934483000000  -0.366576000000
Au   0.001065000000   1.636631000000   1.023103000000
Au   3.350623000000  -1.934483000000  -0.366576000000
10
1.725625000         2
Au  -3.235625000000   0.000000000000  -1.673477000000
Au   0.000000000000   1.296914000000   2.055869000000
Au   0.000000000000  -1.296914000000   2.055869000000
Au  -1.412095000000  -1.371696000000  -0.359799000000
Au   0.000000000000   3.569869000000   0.337206000000
Au   1.412095000000  -1.371696000000  -0.359799000000
Au   1.412095000000   1.371696000000  -0.359799000000
Au  -1.412095000000   1.371696000000  -0.359799000000
Au   0.000000000000  -3.569869000000   0.337206000000
Au   3.235625000000   0.000000000000  -1.673477000000
10
2.385755000         3
Au   1.451179000000   1.350002000000  -0.316516000000
Au   0.000000000000   0.000000000000  -2.481898000000
Au   0.000000000000  -2.654365000000  -2.285364000000
Au  -1.451179000000  -1.350002000000  -0.316516000000
Au  -1.389718000000   0.000000000000   2.013383000000
Au   1.451179000000  -1.350002000000  -0.316516000000
Au   1.389718000000   0.000000000000   2.013383000000
Au  -1.451179000000   1.350002000000  -0.316516000000
Au   0.000000000000   0.000000000000   4.291923000000
Au   0.000000000000   2.654365000000  -2.285364000000
10
3.354615000         4
```

```
Au   0.000000000000   0.000000000000   1.375812000000
Au   0.000000000000   2.364404000000   2.703757000000
Au   0.000000000000  -2.365381000000   0.000000000000
Au   0.000000000000   0.000000000000  -1.375812000000
Au   0.000000000000   0.000000000000  -4.074188000000
Au   0.000000000000   2.365381000000   0.000000000000
Au   0.000000000000  -2.364404000000   2.703757000000
Au   0.000000000000  -2.364404000000  -2.703757000000
Au   0.000000000000   0.000000000000   4.074188000000
Au   0.000000000000   2.364404000000  -2.703757000000
10
3.468820000          5
Au  -0.532087000000  -1.886345000000  -0.362861000000
Au  -2.512031000000  -1.905776000000  -2.141062000000
Au  -2.673962000000  -0.062930000000  -0.281233000000
Au  -0.373531000000   1.569108000000   0.040510000000
Au   4.152544000000  -0.947244000000   0.124871000000
Au   1.710578000000  -1.469939000000   1.063995000000
Au  -0.599572000000  -0.445707000000   1.957774000000
Au   2.186944000000   0.721749000000  -0.495455000000
Au   1.138679000000   2.828732000000  -1.693001000000
Au  -2.497562000000   1.598353000000   1.786461000000
10
3.562945000          6
Au  -0.541994000000   1.018835000000   1.407172000000
Au  -2.926560000000  -0.201854000000   1.394949000000
Au   1.711993000000  -1.777031000000   0.000068000000
Au  -0.541249000000   1.019482000000  -1.407749000000
Au  -2.925956000000  -0.200936000000  -1.394855000000
Au  -0.622224000000  -1.698167000000   1.371902000000
Au   1.943689000000   0.943045000000   0.000456000000
Au  -0.621888000000  -1.697594000000  -1.372705000000
Au   4.105342000000  -0.559971000000   0.000134000000
Au   0.418847000000   3.154191000000   0.000630000000
10
4.304022500          7
Au   0.000000000000   0.000000000000  -1.379663000000
Au   0.000000000000   1.446210000000   0.944271000000
Au   0.000000000000   2.617489000000   3.332434000000
Au   0.000000000000   0.000000000000   3.323114000000
Au   0.000000000000   1.338855000000  -3.804635000000
Au   0.000000000000  -1.338855000000  -3.804635000000
Au   0.000000000000  -2.617489000000   3.332434000000
```

Au 0.000000000000 -2.674306000000 -1.443795000000
Au 0.000000000000 2.674306000000 -1.443795000000
Au 0.000000000000 -1.446210000000 0.944271000000
10
5.737232500 8
Au 1.761127000000 1.335841000000 -0.826160000000
Au 1.761127000000 -1.335841000000 -0.826160000000
Au 0.000000000000 3.443981000000 -0.308406000000
Au 0.000000000000 -3.443981000000 -0.308406000000
Au -1.761127000000 -1.335841000000 -0.826160000000
Au 0.000000000000 -1.379024000000 1.370428000000
Au 0.000000000000 1.379024000000 1.370428000000
Au 0.000000000000 0.000000000000 -2.427915000000
Au -1.761127000000 1.335841000000 -0.826160000000
Au 0.000000000000 0.000000000000 3.608515000000
10
7.359320000 9
Au -1.391220000000 0.798339000000 0.903734000000
Au -0.001450000000 1.680378000000 -1.568494000000
Au -0.001389000000 3.088322000000 0.902539000000
Au -1.341032000000 -0.695259000000 -1.589565000000
Au -3.577895000000 -0.551188000000 0.053286000000
Au 1.342533000000 -0.691964000000 -1.590847000000
Au 3.578158000000 -0.549496000000 0.053589000000
Au -1.299924000000 -1.939512000000 0.965718000000
Au 1.390293000000 0.798778000000 0.905168000000
Au 1.301925000000 -1.938399000000 0.964873000000
10
7.432737500 10
Au -0.429942000000 -0.295999000000 0.000281000000
Au -2.188649000000 1.712886000000 0.000008000000
Au 0.417461000000 2.263446000000 0.000067000000
Au -3.013749000000 -1.115179000000 -0.000053000000
Au 2.966520000000 3.045551000000 0.000195000000
Au 2.540342000000 0.451547000000 0.000015000000
Au -4.755494000000 0.814121000000 -0.000342000000
Au 4.063191000000 -1.732819000000 -0.000427000000
Au 1.484057000000 -2.152757000000 -0.000080000000
Au -1.083736000000 -2.990797000000 0.000335000000
10
7.737075000 11
Au 0.000000000000 3.542498000000 -0.208630000000
Au -1.374060000000 1.332588000000 -1.160426000000

Au -2.402743000000 0.000000000000 1.069132000000
Au 0.000000000000 1.366490000000 1.460349000000
Au 1.374060000000 1.332588000000 -1.160426000000
Au 0.000000000000 -1.366490000000 1.460349000000
Au 1.374060000000 -1.332588000000 -1.160426000000
Au -1.374060000000 -1.332588000000 -1.160426000000
Au 2.402743000000 0.000000000000 1.069132000000
Au 0.000000000000 -3.542498000000 -0.208630000000
10
7.963602500 12
Au -0.489784000000 -1.444657000000 1.375745000000
Au -1.324003000000 -3.561775000000 0.000000000000
Au -0.489784000000 -1.444657000000 -1.375745000000
Au -0.489784000000 1.369448000000 -1.399300000000
Au 1.481989000000 -0.112891000000 2.636255000000
Au 0.997169000000 3.220444000000 0.000000000000
Au 1.481989000000 -0.112891000000 -2.636255000000
Au 1.857626000000 0.523280000000 0.000000000000
Au -2.535635000000 0.194250000000 0.000000000000
Au -0.489784000000 1.369448000000 1.399300000000
10
8.688365000 13
Au -1.400105000000 1.390310000000 0.449826000000
Au -3.674339000000 0.000004000000 0.259369000000
Au 0.000130000000 2.676176000000 -1.531548000000
Au 1.400071000000 1.390250000000 0.449980000000
Au -0.000057000000 -0.000252000000 2.481684000000
Au 1.400079000000 -1.390401000000 0.449629000000
Au -0.000001000000 -2.675863000000 -1.532091000000
Au 3.674274000000 -0.000048000000 0.258926000000
Au 0.000054000000 0.000174000000 -1.735343000000
Au -1.400107000000 -1.390349000000 0.449568000000
10
9.167775000 14
Au 0.000000000000 2.479587000000 -0.812888000000
Au 1.753186000000 3.509005000000 0.832759000000
Au 0.538941000000 1.253691000000 1.563591000000
Au -0.538941000000 -1.253691000000 1.563591000000
Au 0.000000000000 -2.479587000000 -0.812888000000
Au 0.000000000000 0.000000000000 -1.981472000000
Au -1.886450000000 1.775222000000 -2.499855000000
Au 1.886450000000 -1.775222000000 -2.499855000000
Au -1.753186000000 -3.509005000000 0.832759000000

Au 0.000000000000 0.000000000000 3.814258000000
10
9.950267500 15
Au 3.602302000000 0.595900000000 -0.000039000000
Au 4.333585000000 -1.944595000000 -0.000004000000
Au 0.869899000000 1.051057000000 0.000000000000
Au -0.869850000000 -1.051255000000 -0.000095000000
Au -1.728799000000 1.568178000000 -0.000251000000
Au -3.602300000000 -0.595773000000 0.000093000000
Au 2.661693000000 3.034730000000 0.000130000000
Au -2.661934000000 -3.034682000000 -0.000218000000
Au 1.728869000000 -1.568330000000 0.000175000000
Au -4.333464000000 1.944770000000 0.000209000000
10
11.396027500 16
Au -1.453459000000 0.000000000000 1.438930000000
Au -1.405018000000 1.316201000000 -1.021096000000
Au 0.000000000000 -2.336219000000 1.435055000000
Au 1.405018000000 -1.316201000000 -1.021096000000
Au 0.000000000000 2.336219000000 1.435055000000
Au 1.405018000000 1.316201000000 -1.021096000000
Au -1.405018000000 -1.316201000000 -1.021096000000
Au 0.000000000000 -3.730502000000 -0.831793000000
Au 1.453459000000 0.000000000000 1.438930000000
Au 0.000000000000 3.730502000000 -0.831793000000
10
12.795352500 17
Au -3.048210000000 0.808058000000 0.094864000000
Au -0.850144000000 0.980015000000 1.830236000000
Au -2.749527000000 -1.812891000000 -0.488715000000
Au -0.471837000000 -1.610336000000 1.064322000000
Au 1.582342000000 1.185609000000 -1.097274000000
Au 3.827057000000 -0.053921000000 -0.064291000000
Au -0.728095000000 -0.293682000000 -1.465078000000
Au -0.817669000000 2.324586000000 -0.667150000000
Au 1.662136000000 0.055232000000 1.606236000000
Au 1.593948000000 -1.582671000000 -0.813150000000
10
15.235072500 18
Au -1.839724000000 2.531166000000 0.000000000000
Au -2.634683000000 -0.124113000000 0.000000000000
Au 0.689979000000 3.222189000000 0.000000000000
Au 0.000000000000 0.494530000000 0.000000000000

Au 2.631186000000 1.186850000000 0.000000000000
Au -0.361060000000 -2.142160000000 0.000000000000
Au 2.172122000000 -1.412343000000 0.000000000000
Au 3.320741000000 3.711431000000 0.000000000000
Au -1.050627000000 -4.709970000000 0.000000000000
Au -2.927935000000 -2.757578000000 0.000000000000
10
17.553057500 19
Au 0.000000000000 2.324595000000 2.931335000000
Au 0.000000000000 2.385770000000 0.320783000000
Au 0.000000000000 2.435260000000 -2.362752000000
Au 0.000000000000 0.000000000000 1.592109000000
Au 0.000000000000 0.000000000000 -1.288788000000
Au 0.000000000000 -2.385770000000 0.320783000000
Au 0.000000000000 -2.324595000000 2.931335000000
Au 0.000000000000 -4.718835000000 -1.041026000000
Au 0.000000000000 4.718835000000 -1.041026000000
Au 0.000000000000 -2.435260000000 -2.362752000000
10
18.646790000 20
Au -0.780697000000 -2.032893000000 -0.000136000000
Au -0.645896000000 0.643454000000 0.000381000000
Au 1.904634000000 1.992861000000 -0.000075000000
Au -3.163167000000 -0.419048000000 -0.000550000000
Au 1.568938000000 -0.814959000000 -0.000981000000
Au 4.060517000000 0.396906000000 0.000935000000
Au -3.316380000000 -3.021289000000 0.001013000000
Au 3.790268000000 -2.252978000000 -0.000399000000
Au -2.984462000000 2.241314000000 -0.000513000000
Au -0.433754000000 3.266633000000 0.000326000000
10
20.234365000 21
Au -1.421874000000 0.820919000000 0.660321000000
Au 0.000000000000 3.179505000000 0.261996000000
Au 0.000000000000 1.556383000000 -1.893925000000
Au -1.347867000000 -0.778191000000 -1.893925000000
Au 0.000000000000 -1.641839000000 0.660321000000
Au 0.000000000000 0.000000000000 2.914823000000
Au 2.753532000000 -1.589753000000 0.261996000000
Au 1.347867000000 -0.778191000000 -1.893925000000
Au 1.421874000000 0.820919000000 0.660321000000
Au -2.753532000000 -1.589753000000 0.261996000000
10

20.895750000 22
Au 0.136230000000 -1.013334000000 -1.356503000000
Au 0.136230000000 1.821323000000 -1.391432000000
Au 0.136230000000 -1.013334000000 1.356503000000
Au 2.099960000000 0.501354000000 -2.678890000000
Au 2.244059000000 0.415413000000 0.000000000000
Au 2.099960000000 0.501354000000 2.678890000000
Au 0.136230000000 1.821323000000 1.391432000000
Au -1.694256000000 -2.401660000000 0.000000000000
Au -3.742887000000 -3.917595000000 0.000000000000
Au -1.551757000000 3.285156000000 0.000000000000
10
21.487482500 23
Au 0.000000000000 1.387193000000 2.612099000000
Au 0.000000000000 3.606222000000 1.193092000000
Au 0.000000000000 0.000000000000 4.849092000000
Au 0.000000000000 1.338942000000 -0.160570000000
Au 0.000000000000 -1.338942000000 -0.160570000000
Au 0.000000000000 0.000000000000 -2.444042000000
Au 0.000000000000 -1.290624000000 -4.847146000000
Au 0.000000000000 -1.387193000000 2.612099000000
Au 0.000000000000 1.290624000000 -4.847146000000
Au 0.000000000000 -3.606222000000 1.193092000000
10
37.362605000 24
Au -0.481506000000 -3.240397000000 0.000000000000
Au -1.648871000000 -0.882796000000 0.000000000000
Au 1.090357000000 -1.070394000000 0.000000000000
Au 2.636789000000 1.034950000000 0.000000000000
Au 0.895550000000 -5.639588000000 0.000000000000
Au 2.338195000000 -3.488255000000 0.000000000000
Au 0.000000000000 1.449444000000 0.000000000000
Au -2.687954000000 1.597693000000 0.000000000000
Au -1.227563000000 3.857521000000 0.000000000000
Au -0.914997000000 6.381822000000 0.000000000000
